# *Alcohol and Native Language*: Alcohol Use as a Coping Strategy for Intersectional Microaggressions Among Sexual and Gender Minoritized Latine Youth

**DOI:** 10.1080/02791072.2026.2661582

**Published:** 2026-04-23

**Authors:** Aldo Barrita, Roberto L. Abreu, Iván Carbajal, Joshua G. Parmenter, Ryan J. Watson

**Affiliations:** a Department of Psychology, Michigan State University, East Lansing, MI, USA; b Department of Psychology, University of Florida, Gainesville, FL, USA; c School of Psychological Science, Oregon State University, Corvallis, OR, USA; d School of Counseling & Counseling Psychology, Arizona State University, Tempe, AZ, USA; e Department of Human Development and Family Sciences, University of Connecticut, Storrs, CT, USA

**Keywords:** Intersectional microaggressions, SGM Latine youth, bilingual, alcohol use

## Abstract

Intersectional microaggressions – daily slights toward individuals with multiple minoritized identities – pose significant stress for sexual and gender minoritized (SGM) Latine youth. Although research on these experiences has expanded, gaps remain regarding how they influence substance use and mental health across ethnic and linguistic lines. This study examined the relationship between intersectional microaggressions, psychological distress, and alcohol use as a coping strategy among a nationally diverse sample of SGM Latine youth (*N* = 3119). Participants completed surveys assessing experiences of microaggressions, mental health symptoms, and alcohol use. Mediation analyses revealed that intersectional microaggressions (predictor) were associated with higher psychological distress (mediator; *B* = .23, *p* < .001), which in turn were associated with higher alcohol use as a coping mechanism (outcome; *B* = .07, *p* < .001). Psychological distress partially mediated the association between microaggressions and alcohol use (direct effect *B* = .03, *p* < .001). A moderated mediation analysis, in which native language (Spanish or English) was tested as a moderator, showed greater indirect effects for native Spanish speakers than for native English speakers across both indirect paths in the mediation model. Findings underscore the compounded impact of intersectional discrimination on substance use and mental health in SGM Latine youth, with important implications for culturally and linguistically informed interventions targeting minoritized populations.

## Introduction

Substance use during adolescence remains a critical public health concern in the United States (U.S.). Recent data from the Centers for Disease Control and Prevention indicate that one in five adolescents has tried alcohol by age 15 (CDC 2020), and early initiation is linked to long-term negative health and behavioral outcomes (Heradstveit et al. 2017; SAMHSA 2021; Squeglia et al. 2019). Alarmingly, over 90% of adults with substance use disorders report initiating use during adolescence (Centers for Disease Control and Prevention CDC 2020). Among youth, minoritized populations such as *Latine*^1^ adolescents are disproportionately affected, with evidence showing they are nearly twice as likely to consume alcohol by age 18 compared to their non-Latine peers (CDC 2020; SAMHSA 2021). Similarly, sexual and gender minoritized (SGM) youth also report elevated rates of underage drinking, with over half indicating alcohol use by 2021 (The Trevor Project 2021). Despite these disparities, limited research has examined the unique stressors that may contribute to alcohol use in intersectionally minoritized groups, particularly SGM Latine youth. The current study addresses this gap by examining how intersectional microaggressions relate to psychological distress and alcohol use in this population, with attention to potential differences based on native language.

Authors use the term *Latine* to be inclusive of SGM, particularly gender expansive individuals, while acknowledging there is limited research and inconsistent preference among Hispanic, Latino/a/x/e over these panethnic terms.

### Intersectional microaggressions

Intersectional microaggressions – everyday verbal, behavioral, or environmental slights targeting individuals with multiple minoritized identities – can compound stress and limit both external resources (e.g., social support) and internal coping capacities (Abreu et al. 2023; Barrita, Hixson et al. 2023; Barrita, Wong-Padoongpatt et al. 2023; Papa and Parmenter 2025; Parmenter and Barrita 2024; Rosales et al. 2023). Grounded in intersectionality (Crenshaw 1989), which highlights how interlocking systems of oppression uniquely shape the lived experiences of individuals at multiple marginalized intersections, intersectional microaggressions are unique stressors that target and impact groups, such as SGM Latine youth (Rosales et al. 2023).

Similarly, minority stress theory (MST; Meyer 2003) research continues to demonstrate how forms of stress, including intersectional oppression (Parmenter and Barrita 2024), have a direct effect on psychological distress and health outcomes (e.g., depression, anxiety, and substance use) among SGM people of color (Abreu et al. 2025; Parmenter and Barrita 2024; Papa and Parmenter 2025; Zelaya and DeBlaere 2024; Zelaya et al. 2023). Across both frameworks, intersectional microaggressions have been consistently associated with adverse mental health outcomes. Abreu et al. (2023) found that SGM Latine youth frequently encountered microaggressions that invalidated both their cultural background and SGM identities, leading to elevated depressive symptoms. Similarly, Papa and Parmenter (2025) reported that SGM people of color faced microaggressions within SGM and ethnoracial community spaces, which intensified stress and detrimentally affected psychological well-being. Furthermore, intersectional microaggressions have been associated with increased risk for substance use among SGM Latine people (Cerezo et al. 2024; Kler, Shepherd, and Renteria 2023, Zelaya et al. 2023). While these effects have been established, little is known about the mechanisms that may mediate the relations between intersectional microaggressions and the use of alcohol as a coping strategy.

### Intersectional microaggressions, psychological distress, and coping with alcohol

It is important for research with SGM Latine to identify mechanisms at play that influence the association between intersectional microaggressions and alcohol use among SGM Latine youth. The Transactional Model of Stress and Coping (TMSC; Lazarus and Folkman 1984) suggests that individuals assess and respond to stressors based on perceived demands and available coping resources. From this conceptualization, SGM Latine youth may experience heightened psychological distress when facing intersectional microaggressions, which may restrict internal resources for effective coping strategies. Empirical evidence supports this mediational pathway: for instance, DeSon and Andover (2024) found that microaggressions among SGM emerging adults were consistently linked to greater psychological distress, which often functioned as a mediator between minority stress and adverse behavioral outcomes. Similarly, Cerezo and O’Shaughnessy (2021) demonstrated that stigma and discrimination contributed to increased psychological distress among sexually diverse women of color, which in turn was associated with greater alcohol misuse and reduced help-seeking. These findings align with prior work suggesting that psychological distress may serve as a mediator in the association between microaggressions and coping with alcohol (Lewis et al. 2016; Mereish et al. 2025; Rosales et al. 2023), yet only a few recent studies have empirically examined such relationships (Barrita, Hixson et al. 2023; Barrita, Abreu et al. 2025). Furthermore, most research exploring the relations between microaggressions and alcohol use focuses on racial or SGM-based microaggressions rather than intersectional forms (Barrita, Ferraris et al. 2023; Rosales et al. 2023). Scholars have also emphasized the need to examine specific cultural factors and their contributions to such associations, to inform clinical interventions and prevention efforts more effectively (Zelaya et al. 2023).

### Native language differences for Latine youth experiencing oppression

Language plays a vital role in cultural identity for Latine youth. For instance, Spanish proficiency is tied to a connection to Latine culture, and English proficiency is tied to social status among immigrants and their children in the U.S (Zhang et al. 2012). For many Latine individuals with Limited English Proficiency (LEP), additional stressors and difficulties in navigating a new life and culture can lead to acculturative stress. Latine people with LEP may fear victimization or stigma due to language barriers that can directly hinder communication and invoke racial microaggressions. The discomfort and embarrassment can lead to social isolation, diminished self-worth, and psychological distress (Kim et al. 2011). Research, however, shows that Latine individuals who prefer English are more likely to experience physical stress due to racial microaggressions, which is linked to poorer self-rated health outcomes compared to those who prefer Spanish (Gianola et al. 2023). Studies on the impact of limited English proficiency (LEP) among Latine people also suggest that socioeconomic status and acculturation-related factors are more closely tied to psychological distress than LEP (Zhang et al. 2012).

Research often uses language proficiency and LEP as proxies for acculturation (Folsom et al. 2007). Studies on language as a proxy among Latine youth have found a link between alcohol consumption and acculturation levels, with those who are less acculturated being less likely to abuse alcohol than their more acculturated peers (Orona et al. 2007). Other studies have replicated this pattern, showing equivalent results for highly acculturated individuals. For instance, U.S.-born Latine individuals are more likely to struggle with alcohol abuse and dependence, suggesting that embracing drinking norms is a key part of adapting to U.S. culture (Vaeth, Caetano, and Rodriguez 2012). In contrast, Spanish-preferencing adults appear to be partially protected from the negative consequences of stress, independent of other acculturative variables like ethnic background, social relationships, and time in the U.S (Gianola et al. 2023). While these effects around language have been observed in Latine samples, most of these have either used primarily cis-heterosexual samples or have not incorporated differences around gender or sexual identity. Thus, there’s limited understanding of how SGM Latine youth’s experiences with intersectional oppression might differ around native language or how such language is associated with alcohol use, particularly as they face extra stressors stemming from cis-heterosexism. In the current study, native language was examined as a sociocultural factor that may shape stress and coping processes among SGM Latine youth, rather than solely as a proxy for acculturative stress.

### Current study

This study examined the relationship between intersectional microaggressions (experiences of heterosexism and racism), psychological distress, and alcohol use as a coping strategy among SGM Latine youth. We also assessed whether native language (Spanish vs. English) moderated these associations. We hypothesized that (H1) Intersectional microaggressions would be positively associated with alcohol use, and (H2) Psychological distress would mediate the relationship between intersectional microaggressions and alcohol use. No hypotheses were formulated for the moderation analysis, given insufficient evidence to predict specific effects across groups.

## Methods

### Participants and procedures

This online cross-sectional study was conducted following ethical research protocols (masked) and conducted as part of a national SGM teen survey collected in 2022 (masked). This study collected data on SGM youth’s systemic, institutional, and individual experiences and examined how these factors were associated with mental and physical health outcomes in the U.S. Participants were between 13 and 18 years old, resided in the U.S., and identified as sexual and/or gender minority youth (i.e., not heterosexual and cisgender). All measures were presented exclusively in English and included a few attention-check items (e.g., asking participants to select a specific answer). Detailed study information was provided during the informed consent process, and access to the survey was granted only after participants gave consent. Upon completion, participants received a $5 Amazon or Starbucks gift card after their identity was verified through a valid school e-mail or identification card. For the present study, we used responses from participants who identified as Latine or Hispanic. Data is available from the corresponding author upon reasonable request.

### Measures

#### Intersectional microaggressions

The LGBT People of Color Microaggressions Scale (Balsam et al. 2011) was used to assess minority stress by measuring experiences of microaggressions stemming from the intersection of racial/ethnic and sexual/gender identities, comprising 18 items. Example items include, *“People in my racial/ethnic community have told me being gay is a white person’s thing,”* and *“I have felt unwanted in the LGBTQ*+ *community because of my race or ethnicity.”* For each item, participants indicated whether the experience occurred and rated how much it bothered them, using a scale from 1 (not at all) to 5 (almost all the time). Mean scores were computed, with higher scores indicating greater exposure to intersectional microaggressions. This measure showed high reliability in our study (α = .91) and has demonstrated acceptable psychometric properties for diverse minoritized youth (Caba et al. 2024).

#### Psychological distress

The Patient Health Questionnaire (PHQ-4; Kroenke et al. 2009) was used to assess psychological distress; a brief measure evaluating the frequency of depressive and anxiety symptoms experienced over the past 2 weeks. The scale comprises two items assessing depressive symptoms (“Little interest or pleasure in doing things” and “Feeling down, depressed, or hopeless”) and two items assessing anxiety symptoms (“Feeling nervous, anxious, or on edge” and “Not being able to stop or control worrying”). Participants rated each item on a 4-point scale ranging from 0 (*Not at all*) to 3 (*Nearly every day*). Item scores were averaged to produce an overall mean score, with higher scores indicating greater levels of depression and anxiety. The PHQ-4 demonstrated strong internal consistency in the current sample (α = .87) and has been extensively validated across diverse populations (Barrita, Parmenter et al. 2025; Kroenke et al. 2009).

#### Alcohol use as coping strategy

The Drinking Motive Questionnaire – Revised Short Form (DMQ – R SF; Kuntsche and Kuntsche 2009) was used to assess motivations for alcohol use by measuring four primary drinking motives: social, enhancement, coping, and conformity. For this study, participants were asked to indicate how often, in the past 12 months, they consumed alcohol in the following situations: (1) “because it helps you when you feel depressed or nervous,” (2) “to cheer up when you are in a bad mood” (3), “to forget about your problems,” and (4) “to cope with stress.” Responses were rated on a 3-point Likert scale ranging from 0 (*Never*) to 2 (*Almost always*). Mean scores were computed, with higher scores reflecting greater use of alcohol as a coping strategy for distress. The DMQ-R SF demonstrated excellent internal consistency in the current sample (α = .91) and has been efficiently used with diverse minoritized samples (Roberts et al. 2021).

### Native language

Participants were asked about their first language using a categorical variable with options for English, Spanish, or other (with a fill-in-the-box option).

### Demographics

We used a combination of open-ended and multiple-choice questions to assess participants’ demographics. Specifically, participants reported their age and provided information on gender, sexual orientation, and race/ethnicity, selecting all identities that applied. For gender, participants were asked, “*We recognize gender is complex and people may have multiple identities. What is your current gender identity? Select all that apply*,” and sexual orientation was assessed with the question, “*Which of the following describes your sexual orientation?*” See Table 1 for all options.

Ethnicity and race were assessed separately. Participants first indicated whether they identified as Hispanic/Latino/a/x/e (yes/no), which was the only response used to determine qualification for this sub-study. Racial identity was then assessed using multi-categorical options (see Table 1). Participants selecting more than one race were coded as “Multiracial.”

### Analyses

**Figure 1.**
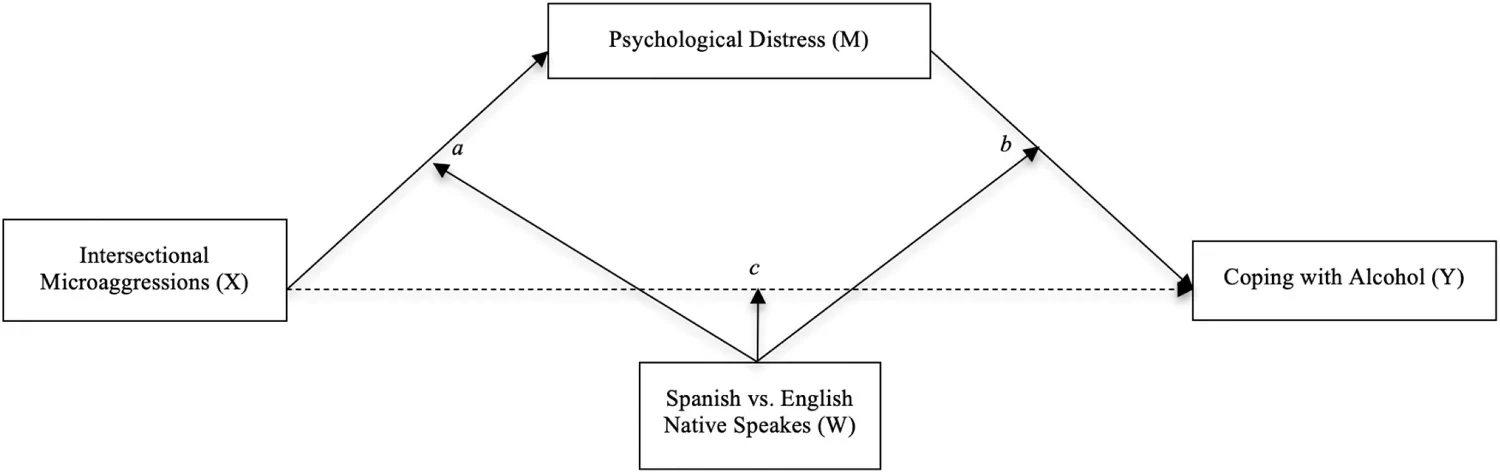
Conceptual model of the relation between intersectional microaggressions and coping using alcohol via psychological distress (mediation), with differences across Spanish and English native speakers.

All analyses were conducted using SPSS Version 29.0. Preliminary checks assessed missing data, assumptions, and outliers. Indirect effects between intersectional microaggressions and alcohol use as a coping strategy via psychological distress were tested using PROCESS Model 4 (Hayes 2012). To test moderating effects, we used PROCESS Model 59 (Hayes 2012), specifying native language (Spanish vs. English) as the moderator (dummy coded as 0 = Spanish, 1 = English). Model 59 tests one moderator across all indirect and direct paths of the mediation (see Figure 1). Both models used 10,000 bootstrap samples to estimate bias-corrected 95% confidence intervals for indirect and conditional effects. Significant interactions were further explored using simple slope analysis. Unstandardized coefficients, standard errors, and 95% confidence intervals are reported for all effects, along with model *R*^*2*^ values and *ΔR*^*2*^ to represent effect sizes and model improvement due to moderation.

## Results

Preliminary analyses examined missing data, attention-check failures, assumptions, and potential covariates. Descriptive statistics and bivariate correlations (see Supplemental Table S1) were reviewed to inform covariate selection. Assumptions for the main analyses, including normality, linearity, independence of errors, and absence of multicollinearity, were assessed using histograms and scatterplots, and no significant violations were found. No significant univariate or multivariate outliers were identified. A small number of participants (1.2%) had missing data on the main study variables. Since this was below 5% and appeared to be missing at random, we used multiple imputation. Additionally, we removed 16 participants for failed attention checks. The final sample (*N* = 3,119; Mage = 15.23, SD = 1.33) was primarily White (38.1%), English-speaking (69.4%), and diverse in gender and sexual identity (see Table 1). A large portion of the sample (31.36%) who did not select a racial identity or selected “Something Else” were categorized as Latine-only based on their initial response to the Hispanic/Latino-ethnicity question. While Latine is an ethnic rather than racial category, this approach accounts for the complex racial and ethnic identities among Latinx populations shaped by colonization and mestizaje, without making assumptions about specific racial identity. With a sample of over 3,000 participants, our study had sufficient power to detect small-to-moderate conditional indirect effects, consistent with recommendations for mediation and moderated mediation analyses (Worthington & Whittaker, 2006).

### Mediation

Mediation analysis results (see Table 2) indicated that intersectional microaggressions were positively associated with higher psychological distress (*B* = 0.23, *SE* = 0.03, 95% CI [0.20, 0.26], *p* < .001). Psychological distress was, in turn, positively associated with alcohol use as a coping strategy, *B* = 0.07, *SE* = 0.01, 95% CI [0.05, 0.08], *p* < .001. The direct effect of intersectional microaggressions on alcohol use as a coping strategy remained statistically significant, *B* = 0.03, *SE* = 0.01, 95% CI [0.01, 0.04], *p* < .01, indicating a partial mediation. The indirect effect of intersectional microaggressions on alcohol use through psychological distress was significant, *B* = 0.02, SE = 0.004, 95% *CI* [0.01, 0.03], *p* < .01. The standardized indirect effect (*β* = .02) indicated a small yet meaningful magnitude. The model accounted for 6.8% of the variance in psychological distress, *R*^*2*^ = .068, *F*(1, 3117) = 227.30, *p* < .001, and 2.4% of the variance in alcohol use as a coping strategy, *R*^*2*^ = .024, *F*(2, 3116) = 38.50, *p* < .001.

### Moderated mediation

Similar to mediation model, moderation mediation findings (see Table 2) showed that intersectional microaggressions were positively associated with psychological distress, *B* = 0.17, *SE* = 0.03, 95% CI [0.11, 0.23], *p* < .001, and with alcohol use as a coping strategy, *B* = 0.03, *SE* = 0.01, 95% CI [0.00, 0.08], *p* < .05. Psychological distress was also positively associated with alcohol use, *B* = 0.22, *SE* = 0.02, 95% CI [0.19, 0.26], *p* < .001.

**Figure 2.**
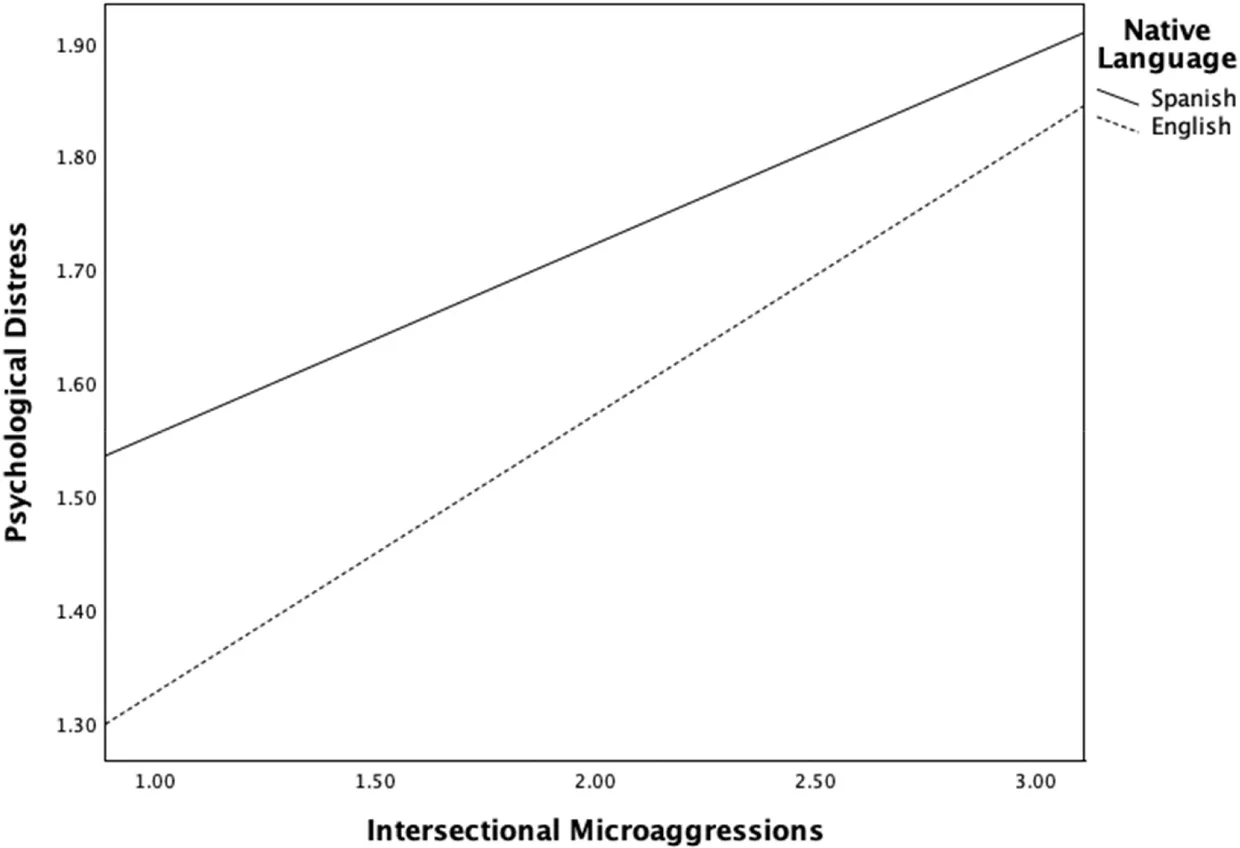
Significant interactions for both Spanish and English native speakers and intersectional microaggressions in relation to psychological distress.

**Figure 3.**
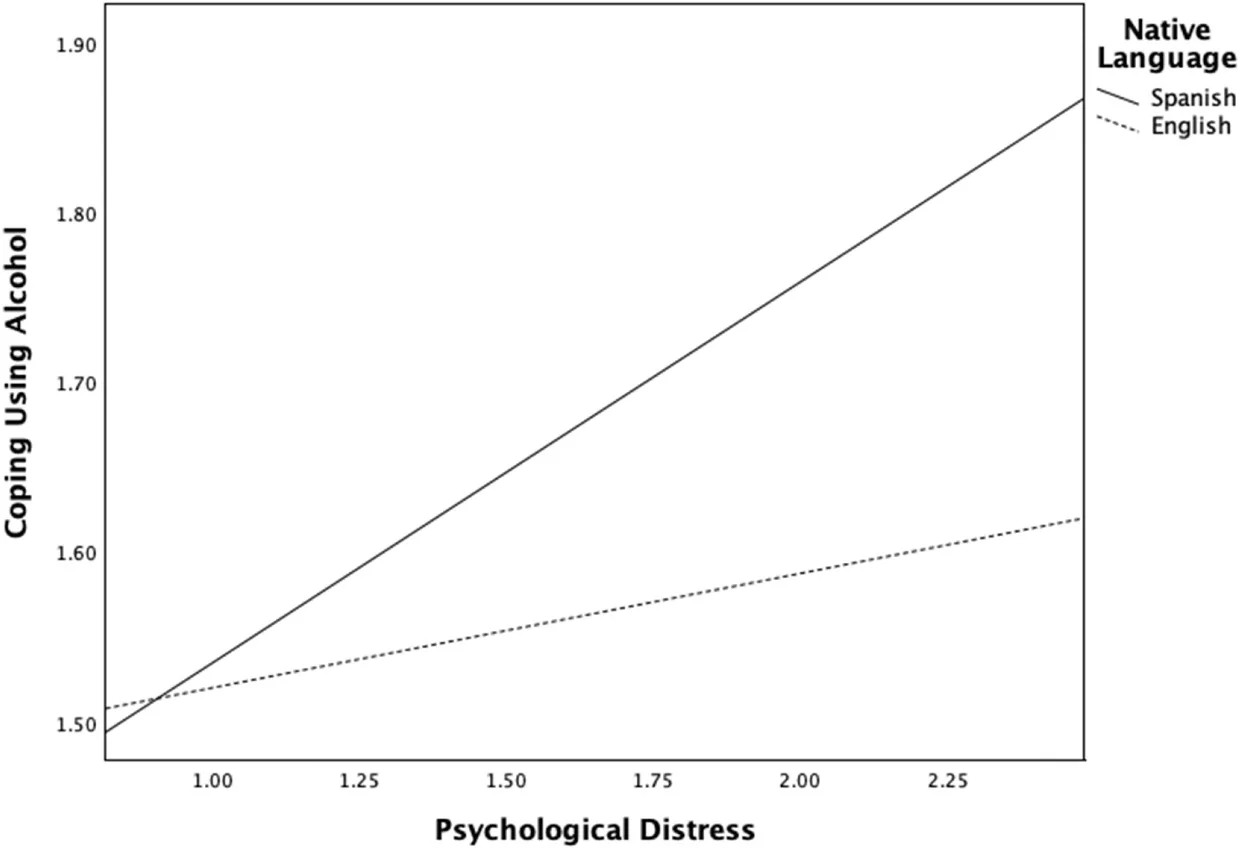
Significant interaction for psychological distress and Spanish native speakers compared to English native speakers in relation to coping with alcohol.

Native language significantly moderated both the path from intersectional microaggressions to psychological distress (*a-path*) and the path from psychological distress to alcohol use (*b-path*). Specifically, the path from intersectional microaggressions to distress was stronger among native Spanish speakers, *B* = 0.08, *SE* = 0.04, 95% *CI* [0.01, 0.15], *p* < .05 (see Figure 2), and the path from distress to alcohol use was also stronger for this group, *B* = 0.16, *SE* = 0.02, 95% *CI* [0.11, 0.21], *p* < .001 (see Figure 3). However, the direct path (*c-path)* between intersectional microaggressions and alcohol use was not moderated based on language group (*p* = .58).

Conditional indirect effects further confirmed moderated mediation, the indirect effect of intersectional microaggressions on alcohol use through psychological distress was significant for both groups but larger among native Spanish speakers (*B* = 0.038, *BootSE* = 0.008, 95% *BootCI* [0.022, 0.056]) than among native English speakers (*B* = 0.017, *BootSE* = 0.004, 95% *BootCI* [0.009, 0.025]). Simple slope analysis (Figures 2 and 3) further depicts these differences in effects. The model accounted for 8.5% of the variance in psychological distress, *R*^*2*^ = .085, F(3, 3115) = 83.58, *p* < .001, and 9.7% of the variance in alcohol use, *R*^*2*^ = .097, *F*(5, 3113) = 59.10, *p* < .001. Adding the interaction terms significantly increased the explained variance for both psychological distress (*ΔR*^*2*^ = .016, *F*(1, 3115) = 4.95, *p* < .05) and alcohol use (*ΔR*^*2*^ = .014, *F*(1, 3112) = 47.87, *p* < .001).

## Discussion

The present study contributes to the growing literature on health disparities among SGM Latine youth by testing a moderated mediation model linking intersectional microaggressions to alcohol use through psychological distress and examining native language as a moderating factor. Consistent with MST and TMSC (Lazarus and Folkman 1984; Meyer 2003), our findings demonstrate that intersectional microaggressions operate as chronic stressors that elevate psychological distress and, consequently, increase the likelihood of alcohol use as a coping strategy. Specifically, SGM Latine youth who reported more experiences of intersectional microaggressions also reported higher levels of distress, which was in turn associated with increased use of alcohol to cope. Although the variance explained for alcohol use was modest, such effect sizes are common in behavioral and minority stress research, where multiple distal and contextual factors contribute to coping behaviors (Funder and Ozer 2019). These findings echo national data showing that early alcohol use among minoritized adolescents, particularly Latine and SGM youth, remains a major public health concern (CDC 2020; SAMHSA 2021; The Trevor Project 2021) and highlights the importance of addressing intersectional forms of discrimination as underlying determinants of health. Although intersectional microaggressions had a direct effect on alcohol use, the indirect pathway through psychological distress accounted for a meaningful portion of this relationship, highlighting distress as a key mechanism, which aligns with prior evidence linking minority stress, psychological distress, and substance use among SGM people of color (Abreu et al. 2023; Rosales et al. 2023; Zelaya et al. 2023).

Our moderated mediation analysis further revealed that these associations were stronger among native Spanish speakers. Compared to English native speakers, native Spanish speakers reported higher psychological distress and greater reliance on alcohol to cope. Notably, the strength of the relationship between intersectional microaggressions and distress, and between distress and alcohol use, was significantly greater in this group. This pattern expands existing acculturation and language research, suggesting that limited English proficiency and linguistic marginalization can compound stress and social exclusion among Latine communities (Kim et al. 2011; Zhang et al. 2012). These results suggest that language background may amplify the psychological and behavioral impacts of discrimination, underscoring the need for culturally and linguistically responsive mental health support. While some prior studies have indicated that English-preferring or more acculturated Latine youth exhibit higher rates of alcohol use (Orona et al. 2007; Vaeth, Caetano, and Rodriguez 2012), our results reveal that for SGM Latine youth, linguistic context intersects with sexual and gender identity to produce unique vulnerability patterns. These differences challenge the notion that Spanish-preferring Latine youth are uniformly protected from stress-related outcomes (Gianola et al. 2023) and instead suggest that the intersection of ethnicity, sexuality, gender, and language intensifies exposure to structural and interpersonal marginalization.

Spanish-speaking SGM Latine youth may navigate compounded exclusion across linguistic, cultural, and queer spaces, encountering microaggressions in predominantly English-speaking SGM environments and in traditional Latine contexts where heteronormative norms persist. This dual marginalization, as intersectionality suggests (Crenshaw 1989), could amplify psychological distress, particularly when youth lack affirming and culturally competent spaces. Further, the dominant SGM discourse in the U.S. is primarily English-speaking and may not offer the culturally resonant language or frameworks that Spanish-speaking youth need to explore and affirm their identities, leaving them more isolated and vulnerable. Moreover, limited access to bilingual and culturally responsive mental health care (APA n.d.) may exacerbate stress exposure, particularly when youth cannot communicate effectively with providers or when providers lack intersectional competence, which can influence increase on reliance of maladaptive coping strategies like alcohol use. Finally, traditional values such as silence, respeto, or familismo in some Spanish-speaking households may discourage help-seeking or open conversations about queerness and mental health, reinforcing internalized stigma and leading youth to self-soothe through substance use.

Our findings suggest several considerations that may be relevant for clinical practice with SGM Latine youth. While the present study did not directly test treatment approaches or prevention programs, the results highlight the potential value of incorporating culturally informed and intersectional frameworks into clinical conceptualization and prevention efforts when working with intersectional minoritized groups, such as SGM Latine youth. For instance, rather than conceptualizing substance use solely as a symptom of psychopathology, an intersectional perspective may help clinicians and prevention specialists recognize how broader systems of discrimination and structural oppression can constrain psychological resources and influence coping behaviors (Arora et al. 2022). Attending to these dynamics may support validation of SGM Latine youths’ experiences of distress and inform discussions of adaptive coping strategies. Existing empirically supported interventions, such as harm reduction approaches (Jenkins, Slemon, and Haines-Saah 2017; Marlatt 1996), mindfulness-based relapse prevention (Witkiewitz, Marlatt, and Walker 2005), and dialectical behavior therapy (Linehan 2014), may offer techniques that align with the need to balance validation of distress with adaptive coping. For example, strategies like distress tolerance, urge surfing, and planning for high-risk situations could be adapted to support youth in reflecting on their values and exploring coping strategies that are meaningful within their cultural and intersectional contexts. Prevention and community-based efforts might also benefit from increasing awareness of intersectional microaggressions and forms of systemic oppression, as creating more affirming environments may help reduce stressors that contribute to health disparities among SGM Latine youth.

Our findings further underscore considerations for working with SGM Latine youth who are native Spanish speakers. There is an ongoing need for bilingual and Spanish-speaking mental health providers (APA n.d.), and training programs could help equip providers with experiences that enhance their capacity to serve these communities. Supporting providers in translating and culturally tailoring interventions, such as substance use prevention and coping strategies, may improve both comprehension and relevance for youth navigating intersectional identities. Frameworks like PARQUE (Causadias and Neblett 2024), which recognize both experiences of oppression and sources of joy, pleasure, and community cohesion, may be useful in informing such efforts.

### Limitations

While our study offers novel evidence of the relation between intersectional microaggressions and alcohol use, several limitations should be noted. The cross-sectional design prevents causal conclusions, though our models are theoretically grounded in prior research (Rosales et al. 2023) that highlights the impact of oppression, which meets experts recommendations in using mediation models with cross-sectional data for minoritized communities that other methods (e.g., longitudinal and experimental) might not be possible to be used in efforts to address social inequities (Hayes and Rockwood 2020). While we used a highly cited and validated scale for intersectional microaggressions (Balsam et al. 2011), more recent adaptations and critiques of this scale have emerged (Caba et al. 2024; Fattoracci, Revels-Macalinao, and Huynh 2021; Huynh et al. 2024), which should be considered when interpreting our results. Furthermore, while several effects reached statistical significance, they accounted for only a modest proportion of the variance, indicating that other unmeasured factors likely influence alcohol coping in this population.

Our approach to native language, despite findings indicating stronger effects for native Spanish speakers, was to conduct all survey materials in English. We did not examine whether intersectional microaggressions occurred in English, Spanish, or both. Similarly, our study asked participants to report their first language, without assessing Latine youth who might have grown up in households where both languages were equally used. Additionally, we did not assess ancestral or national origins, limiting information on racial background for about one-third of the sample. Furthermore, given cultural and linguistic differences across the Spanish-speaking nations, our study did not assess differences in acculturative stressors associated with these variations in language. Future research should focus on creating and validating detailed measures of acculturative stress that consider language and country of origin, especially given the rise of xenophobic narratives targeting Latine communities in recent years. Additionally, measures used for intersectional microaggressions may not fully capture experiences related to skin color or colorism, which have been linked to substance use and other health outcomes among Latinx populations (Cuevas, Dawson, and Williams 2016), highlighting the need for future measures that assess a broader range of intersectional microaggressions. Although concerns exist around alcohol use among Latine youth, we did not assess whether use reached problematic levels. While this study focused on alcohol as a coping mechanism, future research should examine other substances, such as cannabis, opioids, and stimulants, that may also be used to cope with intersectional microaggressions and could have differing prevalence or health impacts in this population. Additionally, while our SGM Latine sample was diverse, we could not examine differences by race, gender, or sexual identity due to small sub-sample sizes. Thus, findings should be interpreted cautiously, and future research should address these gaps.

## Conclusion

This study sheds light on the complex interplay between intersectional microaggressions, psychological distress, and alcohol use among SGM Latine youth, highlighting how structural oppression manifests in individual health behaviors. Our findings make clear that coping with systemic discrimination often unfolds through maladaptive strategies such as substance use, particularly when supportive, culturally affirming, and linguistically accessible resources are scarce. Importantly, we found that native Spanish speakers were especially vulnerable to this pathway, suggesting that language is not simply a demographic variable but a critical axis of marginalization and resilience. As such, interventions must continue to center individual-level treatment and expand to address systemic and structural barriers that perpetuate inequities in care, access, and affirmation. Addressing alcohol use among SGM Latine youth requires public health strategies that recognize how intersectional microaggressions and related psychological distress shape coping behaviors within these communities. By considering the voices and realities of SGM Latine youth, this study reveals how intersectional microaggressions and psychological distress contribute to alcohol coping, calling for future prevention and intervention strategies that move toward equity by addressing these lived stressors.

## Supplementary Material

Supp 1

Supplemental data for this article can be accessed online at https://doi.org/10.1080/02791072.2026.2661582

## Figures and Tables

**Table 1:** Demographic information for sample.

Baseline characteristic	Spanish (*N* = 953)		English (*N* = 2166)	
	*n*	%	*n*	%

Racial Identity				
Alaskan Indian/Native American	119	12.5	0	0.0
Asian	45	4.7	0	0
Black/African American	115	12.1	225	10.4
Pacific Islander	6	0.6	15	0.7
White	188	19.7	998	46.1
Multiracial	53	5.6	314	14.5
Something Else	427	44.8	614	28.3
Sexuality				
Gay or Lesbian	317	33.3	655	30.2
Bisexual	288	30.2	655	30.2
Queer	68	7.1	194	9.0
Pansexual	141	14.8	315	14.5
Asexual	59	6.2	128	5.9
Questioning	27	2.8	115	5.2
Something not listed	53	5.5	106	4.9
Gender Identity				
Cis boy	258	27.1	483	22.3
Cis girl	113	11.9	313	14.5
Transgender girl	51	5.4	115	5.3
Transgender boy	138	14.5	376	17.4
Gender non-conforming	41	4.3	59	2.7
Gender queer	29	3.0	75	3.5
Gender fluid	74	7.8	170	7.8
Nonbinary	134	14.1	304	14.0
Questioning	63	6.6	146	6.7
Something else	52	5.5	124	5.7

* *N* = 3319.

**Table 2.** Mediation and moderated mediation model for the relation between intersectional microaggressions and alcohol use as a coping strategy for discrimination via psychological stress, assessing for differences between Spanish and English native speakers (moderator).

Model 4 Mediation						
	Psychological Distress			Alcohol Use as Coping Strategy		
Predictor	B	SE	95% CI	B	SE	95% CI

Int. Microaggressions (X)	0.233***	.034	0.203: 0.264	0.027**	.008	0.010; 0.043
Psychological Distress (M)				0.066***	.009	0.048; 0.084
Model R^2^	0.068, *F*(1,3117) = 227.3, *p* < .001			0.024, *F*(2,3116) = 38.5, *p* < .001		
Model 59 Moderated Mediation						
Int. Microaggressions (X)	0.168***	.029	0.111; 0.225	0.030*	.007	0.004; 0.084
Psychological Distress (M)				0.224***	.019	0.187; 0.261
Spanish Native Speaker (W)^1^	0.306***	.078	0.153; 0.449	0.167**	.054	0.060; 0.273
X × W	0.078*	.035	0.009; 0.146	−0.012	.021	−0.053; 0.029
M × W				0.157***	.023	0.112; 0.0212
Model R^2^	0.085, *F*(3,3115) = 83.58, *p* < .001			0.097, *F*(5,3113) = 59.1, *p* < .001		
Interaction Δ^2^	0.016, *F*(1,3115) = 4.95, *p* < .05			0.014, *F*(1,3112) = 47.87, *p* < .001		

(*N* = 3119).

1 compared to English Native Speaker.

* *p* < .05.

** *p* < .01.

*** *p* < .001.
