# Supplementary figures and images for: Alcohol and Native Language: Alcohol Use as a Coping Strategy for Intersectional Microaggressions Among Sexual and Gender Minoritized Latine Youth
Source: J Psychoactive Drugs. Author manuscript; Available in PMC 2026 May 27. (PMC13215679; doi:10.1080/02791072.2026.2661582)

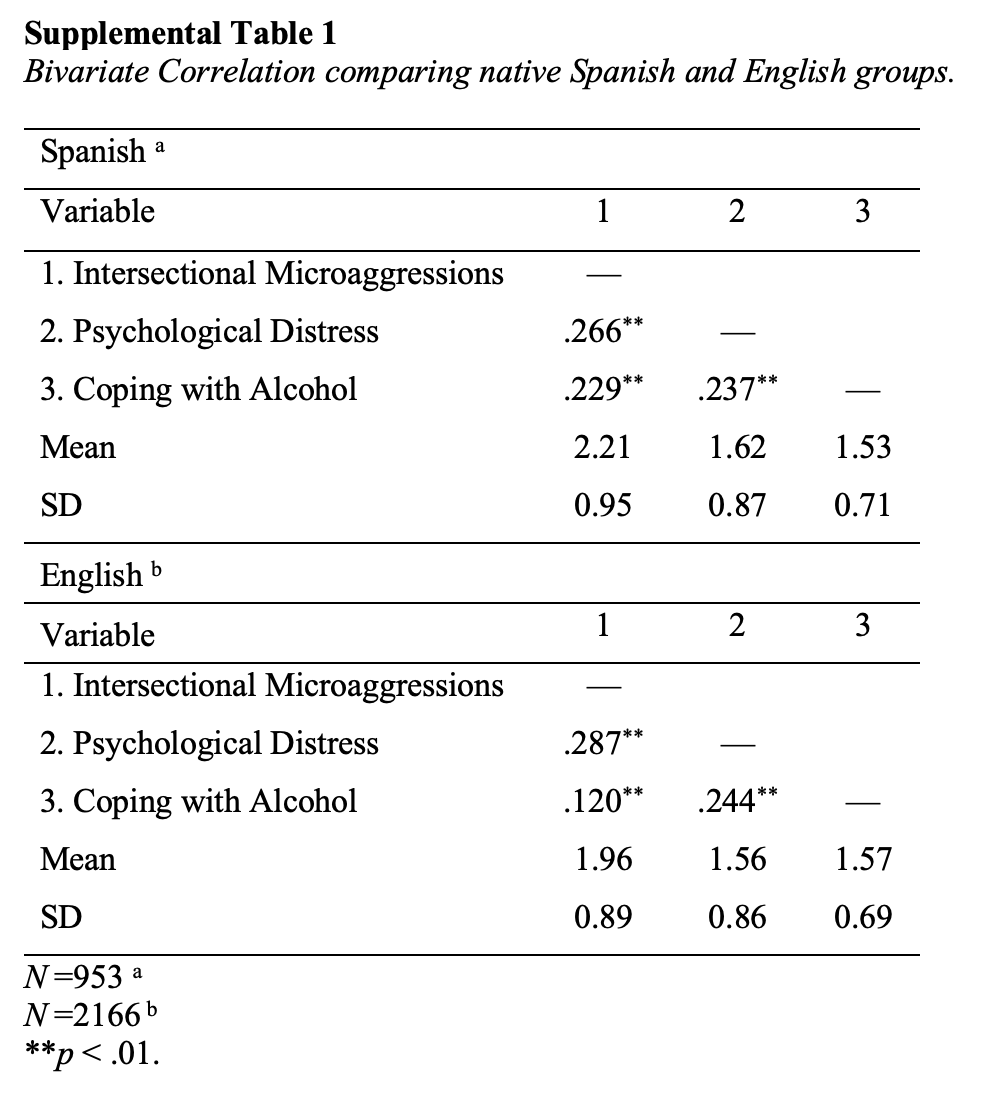

Supplement: Supp 1 [file NIHMS2171343-supplement-Supp_1.docx]
